# Phylogenetic Insights on Patterns of HIV-1 Spread and the Design of Epidemic Control Measures

**DOI:** 10.3390/v14020332

**Published:** 2022-02-07

**Authors:** Bluma G. Brenner

**Affiliations:** McGill Centre for Viral Diseases, Lady Davis Institute, Montreal, QC H3T 1E2, Canada; bluma.brenner@mcgill.ca

Phylogenetics provides a unique structural framework to track the spread of viral diseases, such as HIV-1 (human immunodeficiency virus type 1), the causative agent of AIDS (acquired immunodeficiency syndrome). Viral sequence datasets accrued from national surveillance programs can be analyzed using state-of-the-art bioinformatic tools to deduce genetic interrelatedness, ascertain sequence “clustering” and reconstruct transmission networks. Phylogenetics enhances our understanding of population-level transmission on chronological and stage of infection time scales (Figure 1).

Phylogenetics can be linked with available epidemiological data to ascertain the introduction and spread of viruses in different communities and regional settings. The generalized heterosexual epidemics in Africa and Asia have diversified to include 10 major subtypes (A, B, C, D, F, G, H, J, K) and over 100 circulating recombinant forms (e.g., CRF-1_AE, CRF02_AG). The concentrated epidemics outside of Africa spread unevenly in key groups, including Men having Sex with Men (MSM), People Who Inject Drugs (PWID) and sex worker populations, each influenced by an array of sociodemographic, virological, and behavioral factors.

With no vaccines in the imminent future, the global scale-up of combination antiretroviral therapy treatment is the central pillar of HIV prevention efforts. In 2014, the World Health Organization advanced the 90–90–90 initiative towards epidemic control by 2030. By 2020, all nations were called upon to diagnose 90% of their population, initiate treatment of 90% of those diagnosed, and attain viral suppression for 90% of those treated. The 90–90–90 initiative has reduced transmissions by 38% in sub-Saharan Africa but has fallen short in reaching global infections to 500,000 and 200,000 infections by 2020 and 2030, with 1.7 million new infections in 2020.

To counter the continued rise in infections in the Americas (26% increases in Canada since 2014), the United States (US) Department of Health and Human Services introduced the Ending the HIV Epidemic by 2030 initiative in February 2019. Phylogenetics was added as a fourth pillar to predict clustered outbreaks in 46 jurisdictions and tailor public health responses to reduce infections by 75% and 90% by 2025 and 2030, respectively. Phylogenetic cluster analysis can be integrated with epidemiological demographic and behavioral data to describe underlying factors contributing to the growth of individual epidemics in time and space.

This Special Issue of *Viruses* includes four important articles that highlight the potential role of phylogenetics in describing HIV-1 transmission dynamics in core risk groups in different regional settings [1,2]. The article by Brenner et al. presents leveraged data obtained over an 18-year period (2002 to 2019) to evaluate trends in the spread of subtype B and non-B subtype infections amongst MSM and heterosexual populations in Quebec [1]. The article by Park et al. analyzes contemporary trends in the rise of heterosexual and MSM epidemics in Quebec through migration [2]. The comprehensive review article by Nduva et al uses phylogenetics to compare the endemic spread of HIV-1 among heterosexual and MSM across Africa [3]. The article of Bbosa et al. develops mathematical modelling strategies to ascertain the potential benefit of randomly distributed vs. targeted interventions to avert epidemic growth in different risk groups in Uganda [4].

The studies by Brenner et al. show that half of the HIV infections occurred within small cluster networks (1–5 infections per cluster, median cluster size 2) [1]. Over the 2008 to 2019 period, there were 75% declines in these small networks, concurrent to advancement in treatment-as-prevention paradigms and incentivized testing programs. Unfortunately, epidemic control among MSM was thwarted by the episodic occurrence of large cluster outbreaks averaging 40 persons, expanding over 1- to 5-year intervals. Forty super-spreader variants (5% of viruses) led to micro-epidemics, associated transmission cascades among newly infected, untreated, and younger populations. Large cluster outbreaks among MSM have been observed throughout the world, emphasizing the need to further incentivize pre-exposure prophylaxis, testing and early treatment initiation.

The comprehensive review article by Nduva et al. presents data from 64 phylogenetic studies, describing the differential spread of HIV-1 subtypes and circulating recombinant forms among heterosexual and MSM groups in West and Central Africa, and East and Southern Africa [3]. Cumulative findings reveal that heterosexual HIV epidemics across Africa were largely driven by small cluster networks. In stark contrast, epidemics among MSM from Kenya and Nigeria showed extensive clustering within larger networks. While phylogenetic studies in Africa may be limited by depth of sampling, the lower frequency of clustering and cluster size among heterosexuals may be related to the markedly lower sexual risk per act for heterosexual (vaginal) compared to MSM (anal) and PWID (blood) routes of transmission.

The article by Bbosa et al. compares heterosexual transmission dynamics in three groups in Uganda: the general population in Uganda, including slum areas with high prevalence, high-risk groups of female sexual workers and fisherfolk [4]. Phylogenetics revealed 17% transmission clustering was observed in small, compartmentalized networks. Mathematical modelling approaches were applied to weigh the benefit of generalized random control vs. tailored interventions toward epidemic control. Whereas random control interventions may be of benefit for the general population, targeted interventions are needed to control HIV spread in high-risk fisherfolk communities.

The fastest growing epidemics are among PWID in Eastern Europe and former Soviet-bloc countries. Phylogenetic studies involving PWID in Africa was only reported for Kenya. Africa [3]. Whereas spread among heterosexuals, high-risk sex workers and MSM in coastal Kenya arose through small networks, most PWID sequences were within a single large cluster network showing little overlap with other risk groups. In another Special Issue of *Viruses*, phylogenetics described the dynamics of the introduction and growth of subtype A/E and subtype A1 epidemics among PWID in Bulgaria and Greece, respectively [5,6]. Cluster sizes and transmission rates were significantly higher for PWID than MSM, emphasizing the importance of targeted interventions to avert large outbreaks related to drug use. Notably, Oster et al. showed that epidemics among PWID in the US states are also on the rise, comprising 1% and 11% of clustered infections in 2015–2016 and 2018–2019, respectively [7].

Migration and globalization are leading to a shift towards heterosexual and non-B subtype epidemics in Europe and the Americas. The studies by Brenner et al. and Park et al., illustrated the contemporary role of migration in transmission dynamics [1,2]. The rise in subtype B epidemics among heterosexuals and MSM (25% of cases) in Quebec has arisen through migration from Haiti, and other countries in the Caribbean, Central and South America. Non-B subtype epidemic represent recent migration from countries in West and Central Africa in francophone countries. In 2017, recent migrants have risen to 54% of new provincial infections. Clearly, targeted public health programs are needed to assure that new arrivals have access to treatment and prevention interventions to avert the acquisition and spread of HIV.

Taken together, these articles show significant progress occurring in sub-Saharan Africa despite weak public health infrastructures. Clearly, the scale-up of access to antiretroviral therapy, including integrase inhibitor-based regimens has been of incredible benefit. Epidemic control will require the best available antiviral drugs to sustain long-term viral suppression, prevent emergent drug resistance, and avert secondary outbreaks.

Global control of MSM and PWID epidemics have lagged. Phylogenetics reveal the importance for tailored interventions for MSM and PWID to target those who are most affected, following legal and ethical imperatives.

## Figures and Tables

**Figure 1:**
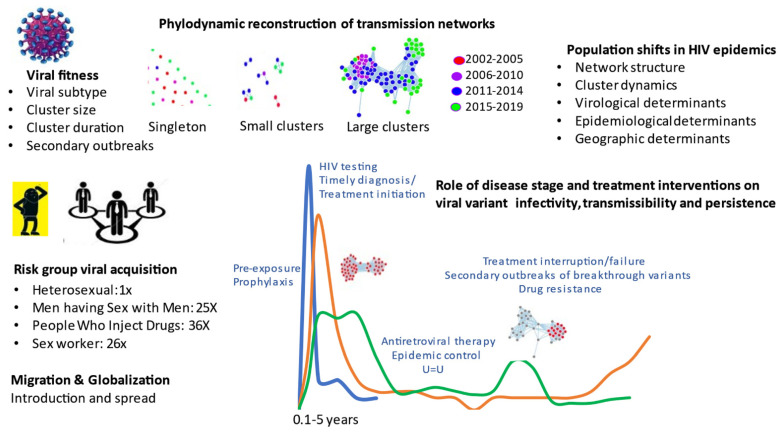
Phylogenetics inferences in HIV transmission towards epidemic control.

## Data Availability

Sequences from the Quebec provincial genotyping program cannot be made available for confidentiality purposes.
